# Primary Hyperparathyroidism in a Pregnant Immigrant Facing Healthcare Hurdles: A Case Report

**DOI:** 10.1155/crog/7503619

**Published:** 2026-06-15

**Authors:** Racquel McCrary, Tina Huang, Miranda Worley, Rebecca Daugherty, Amanda Stisher

**Affiliations:** ^1^ University of Alabama at Birmingham Marnix E. Heersink School of Medicine, Huntsville Campus, Birmingham, Alabama, USA

**Keywords:** case report, fetal hydrops, pregnancy, primary hyperparathyroidism, social determinants of health

## Abstract

**Introduction:**

PHPT in pregnancy is a rare condition that may be difficult to recognize due to nonspecific symptoms and physiologic changes that can mask hypercalcemia. Delayed diagnosis is associated with significant maternal and fetal morbidity.

**Case Presentation:**

A 36‐year‐old Spanish‐speaking gravida 5 para 3 woman at 30 weeks gestation presented with decreased fetal movement in the setting of absent prenatal care. Evaluation revealed severe polyhydramnios, fetal growth restriction, and nonimmune fetal hydrops. Laboratory studies demonstrated hypercalcemia with elevated parathyroid hormone levels, consistent with PHPT, with preserved renal function and concurrent vitamin D insufficiency. Ultrasound identified a parathyroid adenoma. Initial management with intravenous hydration and calcitonin resulted in only partial improvement in calcium levels. Ongoing management was complicated by competing priorities between treatment of maternal hypercalcemia and obstetric concerns, as escalation of fluid therapy was limited by the risk of fluid overload in the setting of severe polyhydramnios. Despite medical management, worsening fetal status, including abnormal umbilical artery Doppler findings, necessitated preterm cesarean delivery. The neonate required intubation and admission to the neonatal intensive care unit for management of hydrops fetalis. Following delivery, the patient underwent definitive surgical management with parathyroidectomy, resulting in normalization of calcium and parathyroid hormone levels and confirming biochemical cure.

**Conclusion:**

This case highlights the diagnostic challenges of PHPT in pregnancy and the complexity of management when standard treatment pathways are limited. It also demonstrates how delayed access to prenatal care and language discordance may contribute to more advanced disease at presentation. Early recognition and coordinated multidisciplinary care may improve maternal and fetal outcomes. This case supports consideration of PHPT in pregnant patients with hypercalcemia and informs clinical approaches to management in complex presentations.

## 1. Introduction

Primary hyperparathyroidism (PHPT) in pregnancy is a rare endocrine disorder, with an estimated incidence of approximately 1%. It is typically diagnosed in the setting of hypercalcemia with an inappropriately normal or elevated parathyroid hormone (PTH) level [1].

PHPT in pregnancy is associated with significant maternal and fetal morbidity. Maternal complications include hyperemesis gravidarum, nephrolithiasis, pancreatitis, hypertension, and hypercalcemic crisis. Fetal and neonatal complications include miscarriage, preterm birth, intrauterine growth restriction (IUGR), neonatal hypocalcemia, and fetal demise. Early recognition is essential to reduce the risk of adverse outcomes.

Importantly, social determinants of health, including immigration‐related barriers, financial instability, limited social support, and language discordance, may restrict access to prenatal care, resulting in delayed diagnosis and more advanced disease at presentation.

This manuscript was prepared following the CARE guidelines (https://www.care-statement.org/).

## 2. Case Report

A 36‐year‐old Hispanic, Spanish‐speaking gravida 5 para 3 woman at 30 weeks and 4 days′ gestation presented to the obstetric emergency department with decreased fetal movement. She had no significant past medical history, took no medications, and was a never smoker. Her obstetric history included three prior uncomplicated vaginal deliveries outside of the United States.

She had received no prenatal care during this pregnancy due to financial instability and lack of a support system. Interpreter services were required throughout her hospitalization. Her immigration status was not documented.

Prior to presentation, she underwent a nonmedical boutique ultrasound and was advised that she had excessive amniotic fluid and should seek medical evaluation. She delayed the presentation until the end of a holiday weekend due to uncertainty regarding clinic availability. At an outside hospital, ultrasound demonstrated an amniotic fluid index (AFI) of 52 cm, and she was transferred for further evaluation.

On admission, repeat ultrasound by maternal‐fetal medicine (MFM) confirmed severe polyhydramnios with an AFI of 44.6 cm and updated gestational age to 31 weeks and 4 days. Additional findings included persistent absent umbilical artery end‐diastolic flow and multiple fetal anomalies, including micrognathia, ventricular septal defect, bilateral femur measurements below the 1st percentile, clenched hands, and nonimmune hydrops fetalis. She was diagnosed with polyhydramnios, chronic hypertension with concern for superimposed preeclampsia versus chronic proteinuria, and fetal growth restriction. Laboratory findings are summarized in Table 1.

**Table 1:** Initial laboratory findings.

Laboratory test	Value	Reference range
Total calcium	12.4 mg/dL	8.6–10 mg/dL
Ionized calcium	1.63 mmol/L	1.10–1.35 mmol/L
Parathyroid hormone (PTH)	192 pg/mL	15–65 pg/mL
Creatinine	0.9 mg/dL	0.5–1.0 mg/dL
Blood urea nitrogen (BUN)	9 mg/dL	6–20 mg/dL
EGFR	85 mL/min/1.73 m^2^	> 60
Albumin	3.0 g/dL	3.5–5.2 g/dL
25‐Hydroxyvitamin D	27.6 ng/mL	30–80 ng/mL
Phosphorus	Not obtained on admission	2.7–4.5 mg/dL
Magnesium	Not obtained on admission	1.6–2.4 mg/dL

Endocrinology was consulted and suspected PHPT given the elevated calcium and PTH levels, along with a history of nephrolithiasis that was later elicited from the patient. Urology was consulted after the patient passed a renal stone during hospitalization. Renal ultrasound demonstrated bilateral medullary nephrocalcinosis and nephrolithiasis, as well as cholelithiasis.

Initial management included intravenous normal saline bolus followed by continuous infusion at 100 cc/h and initiation of vitamin D supplementation at 50,000 units weekly due to vitamin D insufficiency. After minimal improvement in calcium levels (12.4–11.5 mg/dL), an additional 1 L normal saline bolus was administered and the infusion rate increased to 125 cc/h. Calcitonin therapy (4 mg/kg every 12 h) was initiated as adjunctive treatment. Calcitonin was selected due to its minimal placental transfer, despite its limited efficacy and safety data in pregnancy.

Localization imaging with sestamibi scan and computed tomography (CT) was initially considered but deferred during pregnancy due to fetal radiation exposure risk. Consequently, surgical intervention was postponed to the postpartum period. A thyroid ultrasound was performed and demonstrated a 2.1 cm heterogeneous, hypervascular nodule abutting the inferior pole of the right thyroid lobe, consistent with a parathyroid adenoma in the setting of hypercalcemia.

Following escalation of intravenous fluids and calcitonin, calcium improved to 10.6 mg/dL and ionized calcium to 1.55 mmol/L, prompting discontinuation of calcitonin. However, management was complicated by competing priorities between endocrinology and obstetrics. Although endocrinology recommended increasing intravenous fluids to 150 cc/h for hypercalcemia management, obstetrics reduced the rate to 100 cc/h due to concern for fluid overload in the setting of severe polyhydramnios. Calcium levels remained stable during this period, and fluids were subsequently increased again to 150 cc/h.

Due to worsening fetal status, including fetal growth restriction and progression from absent to intermittent reversed end‐diastolic flow on umbilical artery Doppler, MFM recommended delivery at 32 weeks and 2 days′ gestation. The patient underwent a cesarean section with bilateral salpingectomy. A live female infant weighing 1660 g was delivered with Apgar scores of 3, 6, and 8. The neonate required intubation and was admitted to the neonatal intensive care unit for management of hydrops fetalis. Neonatal calcium levels were not available in the medical record.

Immediately prior to delivery, calcium level was 11.1 mg/dL, and endocrinology recommended reducing intravenous fluids to 100 cc/h due to concern for pulmonary edema after several days of high‐rate fluid administration for hypercalcemia management. Calcitonin therapy was resumed given persistent hypercalcemia. Following delivery, calcium decreased to 10.1 mg/dL.

Postpartum CT imaging demonstrated a homogeneously enhancing nodule measuring 3.1 × 1.5 cm at the inferior margin of the right thyroid lobe. Three days later, the patient underwent a parathyroidectomy. The resected adenoma measured 3.0 × 2.0 × 1.6 cm and weighed 2.6 g. Intraoperative PTH decreased from 101 pg/mL to 36.4 pg/mL, consistent with biochemical cure.

Postoperative laboratory values demonstrated normalization of calcium and PTH levels (Table 2). The patient was started on calcium carbonate 1250 mg three times daily, and intravenous fluids were discontinued.

**Table 2:** Postoperative Day 1 lab values.

Laboratory test	Value	Reference range
Total calcium	9.0 mg/dL	8.6–10 mg/dL
Ionized calcium	1.18 mmol/L	1.10–1.35 mmol/L
Parathyroid hormone (PTH)	16.2 pg/mL	15–65 pg/mL
Phosphorus	5.6 mg/dL	2.5–4.5 mg/dL
Magnesium	1.6 mg/dL	1.6–2.4 mg/dL

Her postoperative course was notable for transient urinary retention and ongoing nephrolithiasis. She was discharged in stable condition with outpatient follow‐up arranged with obstetrics, endocrinology, otolaryngology, and urology.

## 3. Discussion

PHPT is a rare endocrine disorder in pregnancy, with an estimated incidence of approximately 1% [2]. Despite its low prevalence, PHPT can result in significant maternal and fetal morbidity if left untreated. True prevalence may be underestimated due to diagnostic challenges.

Maternal complications include nephrolithiasis, pancreatitis, bone disease, hypertension, pre‐eclampsia, and hypercalcemic crisis. Fetal complications include neonatal hypocalcemia, miscarriage, stillbirth, IUGR, preterm birth, seizures, hypotonia, respiratory distress, and low birth weight [2, 3]. Due to the potential severity of PHPT, prompt diagnosis and management are essential.

PHPT is diagnosed by elevated serum ionized calcium or albumin‐corrected calcium in the setting of an elevated or nonsuppressed PTH [1]. Serum calcium levels are not routinely monitored during pregnancy, requiring a high index of suspicion for diagnosis. PHPT symptoms include nausea, vomiting, weakness, fatigue, headache, and neurobehavioral changes [2]. These symptoms frequently overlap with normal pregnancy, which may delay further evaluation. Additionally, physiologic changes during pregnancy may mask hypercalcemia and further delay diagnosis. Serum calcium levels in patients with PHPT may not reach elevated levels due to physiologic lowering of serum calcium in pregnancy due to hypoalbuminemia, increased glomerular filtration, and transplacental transfer of calcium [3, 4].

Although many patients may be asymptomatic, symptomatic presentations or incidental laboratory findings should prompt further evaluation [2]. The most common cause of PHPT in pregnancy is a parathyroid adenoma, which can often be identified with ultrasound. Imaging with 99mTc‐sestamibi scintigraphy is avoided during pregnancy due to fetal radiation exposure [3]. Other causes of PHPT include genetic conditions such as multiple endocrine neoplasia (MEN) syndromes [1]. In our patient, the presence of a solitary adenoma and lack of reported family history made MEN syndrome less clinically suspected; however, genetic evaluation was deferred because acute maternal and fetal management took precedence [5]. Once PHPT is suspected, identifying the underlying etiology is essential to guide management.

Management of PHPT in pregnancy is individualized, as there is no universal consensus on treatment [1]. Conservative management includes intravenous hydration, calcium restriction, and vitamin D supplementation [3]. Calcitonin may be used for refractory hypercalcemia due to its minimal placental transfer, whereas cinacalcet crosses the placenta and has limited safety data in pregnancy [1]. In our case, serum calcium levels did not significantly lower with conservative management so calcitonin was added as adjunctive therapy.

Definitive management of symptomatic PHPT due to parathyroid adenoma is surgical resection, which is typically recommended during the second trimester [6]. Surgery in the third trimester has been reported but carries increased maternal and fetal risk. Parathyroidectomy is generally recommended when serum calcium levels exceed 11 mg/dL due to increased risk of complications [2]. Our patient presented in the third trimester, outside of the optimal window for parathyroidectomy. Parathyroidectomy was initially planned before delivery; however, worsening fetal status necessitated delivery before surgical intervention.

Previous reports have described earlier diagnosis and management of PHPT in pregnancy, often allowing for second‐trimester parathyroidectomy or successful conservative management [7]. In contrast, our patient presented later in pregnancy with severe hypercalcemia, polyhydramnios, and fetal complications such as fetal anomalies and fetal hydrops, limiting the feasibility of second‐trimester surgical intervention. This highlights the challenges of managing PHPT in late pregnancy, particularly when maternal treatment priorities conflict with obstetric indications for delivery.

Shani et al. [7] proposed a potential association between maternal hypercalcemia and polyhydramnios, suggesting that fetal exposure to elevated calcium levels may lead to polyuria and increased amniotic fluid volume. This mechanism is thought to resemble nephrogenic diabetes insipidus, where impaired renal concentrating ability results in excessive fetal urine production.

Polyhydramnios has also been observed to coexist with nonimmune fetal hydrops, likely due to disruption in fetal fluid regulation and interstitial fluid balance [8]. However, a direct causal relationship between PHPT and fetal hydrops has not been well established. In this case, severe maternal hypercalcemia may have contributed to polyhydramnios and subsequent fetal compromise, representing a potential contributing factor to the development of nonimmune fetal hydrops.

This case highlights the role of social determinants of health influencing disease severity and clinical outcomes. Limited access to prenatal care likely contributed to the delayed diagnosis of PHPT, whereas language discordance necessitated reliance on interpreter services and may have impacted care coordination [9]. Prior studies have demonstrated that language discordance is associated with increased patient confusion, frustration, and perceived poor quality of care [10]. These factors emphasize the importance of improving access to prenatal care and language‐concordant services in the management of complex conditions during pregnancy.

## 4. Conclusion

This case highlights key considerations in the evaluation and management of PHPT in pregnancy. PHPT should be considered in pregnant patients with hypercalcemia, even when symptoms are nonspecific or attributed to normal pregnancy. Physiologic changes may mask laboratory abnormalities, contributing to delayed diagnosis and increasing the risk of maternal and fetal complications.

Management can be challenging when standard treatment pathways are limited, such as when intravenous hydration and calcitonin provide only temporary control of hypercalcemia, or when definitive surgical management is not feasible within the optimal timeframe. Concurrent obstetric complications, including polyhydramnios and fetal compromise, can further complicate management and influence the timing of delivery and intervention.

This case also demonstrates how social determinants of health, including limited access to prenatal care and language discordance, may contribute to delayed presentation, advanced disease at diagnosis, and challenges in care coordination. Overall, this case underscores the importance of early recognition, multidisciplinary management, and consideration of both clinical and social factors when caring for pregnancies complicated by PHPT.

## Author Contributions

Racquel McCrary affirms that this manuscript is an honest, accurate, and transparent account of the study being reported; that no important aspects of the case have been omitted; and that any discrepancies from the study as planned have been explained.

## Funding

No funding was received for this manuscript.

## Disclosure

All authors have read and approved the final version of the manuscript. The corresponding author had full access to all of the data in this study and takes complete responsibility for the integrity of the data and the accuracy of the data analysis.

## Ethics Statement

All authors attest they meet current ICMJE criteria for authorship.

## Consent

Informed consent to publish has been obtained.

## Conflicts of Interest

The authors declare no conflicts of interest.

## Data Availability

Data sharing not applicable to this article as no datasets were generated or analyzed during the current study.

## References

[1] Ali D. S. , Dandurand K. , and Khan A. A. , Primary Hyperparathyroidism in Pregnancy: Literature Review of the Diagnosis and Management, Journal of Clinical Medicine. (2021) 10, no. 13, 10.3390/jcm10132956, 34209340. PMC8268799 34209340

[2] Alimena S. , Razvi K. , and Morosky C. M. , Primary Hyperparathyroidism Diagnosed During the Work-Up of Hydrops Fetalis: A Case Report, International Journal of Obstetrics and Gynaecology Research. (2016) 3, no. 9, 474–480.

[3] Kamenicky P. , Lecoq A. L. , and Chanson P. , Primary Hyperparathyroidism in Pregnancy, Annales d′Endocrinologie. (2016) 77, no. 2, 169–171, 10.1016/j.ando.2016.04.010. 27157105

[4] Malekar-Raikar S. and Sinnott B. P. , Primary Hyperparathyroidism in Pregnancy—A Rare Cause of Life-Threatening Hypercalcemia: Case Report and Literature Review, Case Reports in Endocrinology. (2011) 2011, 520516, 10.1155/2011/520516. 22937284 PMC3420708

[5] McCarthy A. , Howarth S. , Khoo S. , Hale J. , Oddy S. , Halsall D. , Fish B. , Mariathasan S. , Andrews K. , Oyibo S. O. , Samyraju M. , Gajewska-Knapik K. , Park S. M. , Wood D. , Moran C. , and Casey R. T. , Management of Primary Hyperparathyroidism in Pregnancy: A Case Series, Endocrinology, Diabetes & Metabolism Case Reports. (2019) 2019, 10.1530/EDM-19-0039, 31096181. PMC6528402 31096181

[6] Pliakos I. , Chorti A. , Moysidis M. , Kotsovolis G. , Kaltsas T. , Pana A. , Ioannidis A. , and Papavramidis T. S. , Parathyroid Adenoma in Pregnancy: A Case Report and Systematic Review of the Literature, Frontiers in Endocrinology. (2022) 13, 975954, 10.3389/fendo.2022.975954, 36325457. 36325457 PMC9618884

[7] Shani H. , Sivan E. , Cassif E. , and Simchen M. J. , Maternal Hypercalcemia as a Possible Cause of Unexplained Fetal Polyhydramnion: A Case Series, American Journal of Obstetrics and Gynecology. (2008) 199, no. 4, 10.1016/j.ajog.2008.06.092. 18928992

[8] Vanaparthy R. , Vadakekut E. S. , and Mahdy H. , Nonimmune Hydrops Fetalis, in Stat Pearls, 2026, StatPearls Publishing. 33085361

[9] Camargo J. T. , Barral R. L. , Kerling E. H. , Saavedra L. , Carlson S. E. , Gajewski B. J. , and Ramírez M. , Prenatal Care Utilization Challenges and Facilitators for a Growing Latino Community in the Midwest, Maternal and Child Health Journal. (2023) 27, no. 10, 1811–1822, 10.1007/s10995-023-03733-1, 37369811. 37369811 PMC11251489

[10] Escobedo L. E. , Cervantes L. , and Havranek E. , Barriers in Healthcare for Latinx Patients With Limited English Proficiency—A Narrative Review, ournal of General Internal Medicine. (2023) 38, no. 5, 1264–1271, 10.1007/s11606-022-07995-3, 36720766. PMC9888733 36720766

